# Isolation and preliminary characterization of a glucan-type exopolysaccharide produced by *Bacillus tequilensis* strain HH from Egyptian fermented cucumber

**DOI:** 10.1038/s41598-026-62841-4

**Published:** 2026-07-30

**Authors:** H. R. Heba, M. M. Amer, Mahmoud M. A. Elsayed, Aly E. Abo-Amer

**Affiliations:** 1 Sohag Regional Blood Transfusion Center, Sohag, Egypt; 2 Department of Botany and Microbiology, Faculty of Science, Benha University, Benha, Egypt; 3 Department of Pharmaceutics and Clinical Pharmacy, Faculty of Pharmacy, Sohag University, Sohag, 82524 Egypt; 4 Department of Botany and Microbiology, Faculty of Science, Sohag University, Sohag, 82524 Egypt

**Keywords:** *Bacillus tequilensis*, *Bacillus subtilis* species complex, glucan-type exopolysaccharide, FTIR, NMR, cytocompatibility, Biochemistry, Biological techniques, Biotechnology, Microbiology

## Abstract

Exopolysaccharide (EPS) production is a common feature of microbial carbohydrate metabolism during fermentation; however, glucan-type EPS produced by *Bacillus* species in fermented vegetable systems remain insufficiently characterized. This study aimed to isolate an EPS-producing bacterium from naturally fermented cucumber and to perform a preliminary physicochemical and spectroscopic characterization of the produced polymer. An EPS-producing bacterial isolate was recovered and identified as *Bacillus tequilensis*, a member of the *Bacillus subtilis* species complex, based on 16 S rRNA gene sequence analysis. The extracellular polysaccharide was partially purified and analyzed using FTIR and ¹H NMR spectroscopy, revealing structural features consistent with a glucan-type EPS. Rheological analysis demonstrated a concentration-dependent increase in viscosity, confirming the macromolecular nature of the polymer in aqueous systems. Sucrose-hydrolyzing activity was detected under optimized conditions, indicating active carbohydrate metabolism associated with EPS production. In vitro cytocompatibility assessment using Vero and WI-38 cell lines showed high cell viability at low to moderate concentrations, with a dose-dependent reduction observed at higher levels. Overall, this study reports the isolation of an EPS-producing *Bacillus tequilensis* strain from Egyptian fermented cucumber and provides preliminary insights into the properties of its glucan-type exopolysaccharide, highlighting fermented vegetable microbiota as a potential source of functional biopolymers.

**Supplementary Information:**

The online version contains supplementary material available at 10.1038/s41598-026-62841-4.

## Introduction

Microbial exopolysaccharides (EPS) are high-molecular-weight carbohydrate polymers secreted into the extracellular environment, where they contribute to cell adhesion, protection against environmental stress, and biofilm formation^1,2^. Owing to their biodegradability, structural diversity, and reported biocompatibility in various systems, EPS have attracted increasing interest in food, pharmaceutical, and biotechnology-related fields^3^. Based on monosaccharide composition, EPS are generally classified into homopolysaccharides, composed of a single type of monosaccharide, and heteropolysaccharides consisting of repeating units of different sugars and sugar derivatives^4,5^.

Among microbial homopolysaccharides, glucan-type polymers have been extensively investigated due to their distinctive solution behavior and functional versatility. Dextran is one of the most widely studied microbial glucans and is predominantly composed of α-linked glucopyranose units, exhibiting characteristic physicochemical properties^6^. Glucan-type EPS production has been most frequently associated with lactic acid bacteria (LAB), particularly *Leuconostoc* species, although similar polymers have also been reported in other bacterial genera, including *Lactobacillus*, *Weissella*, and certain *Bacillus* species^7,8^.

Beyond their traditional role as food stabilizers and texturizing agents, microbial EPS have been explored for a range of functional applications^9,10^. Comprehensive structural and physicochemical characterization is essential to accurately define structure–property relationships and to evaluate the functional potential of newly identified polymers^11,12^.

LAB is among the most extensively studied producers of EPS in fermented foods due to their long-standing use and recognized safety status^13,14^. In fermented vegetable systems, LAB contributes to texture development and product stability through carbohydrate metabolism and extracellular polysaccharide formation^15,16^. However, these ecosystems also harbor diverse non-lactic, Gram-positive bacteria that may participate in extracellular polymer production under specific environmental and nutritional conditions^17^.

Members of the *Bacillus subtilis* species complex are recognized for their metabolic versatility, favorable safety profiles, and capacity to synthesize various extracellular biopolymers^18,19^. Compared with LAB-derived EPS, reports describing glucan-type exopolysaccharide production by *Bacillus* species isolated from fermented vegetables remain limited, and the physicochemical and biological properties of such polymers have not been comprehensively characterized^20,21^. Although *Bacillus* species are more frequently associated with the production of heteropolysaccharides, levan, or γ-polyglutamic acid^22,23^, certain strains have been reported to produce sucrose-dependent extracellular polysaccharides under defined culture conditions^24,25^.

Nevertheless, the occurrence and preliminary characterization of glucan-type exopolysaccharide production by members of the *Bacillus subtilis* species complex in fermented vegetable systems remain insufficiently documented^26^. Although recent studies have reported glucan-type exopolysaccharides produced by *Bacillus* with diverse physicochemical and structural properties, such investigations have primarily focused on non-vegetable fermentation systems. Despite increasing interest in microbial exopolysaccharides, data on glucan-type EPS production by *Bacillus* species specifically within vegetable fermentation ecosystems remains limited^27^.

Therefore, the aim of the present study was to isolate and identify an EPS-producing bacterial strain from naturally fermented cucumber and to perform a preliminary physicochemical and spectroscopic characterization of the produced exopolysaccharide. In addition, selected rheological properties and in vitro cytocompatibility were evaluated to provide initial insight into the functional potential of the recovered polymer.

## Materials and methods

The following experimental procedures were employed to isolate and characterize exopolysaccharide (EPS)-producing bacteria associated with naturally fermented cucumber samples. An EPS-producing bacterial isolate was recovered and subjected to subsequent phenotypic and molecular identification (Sect. 2.3). Culture-based enrichment and isolation strategies commonly applied for fermentative bacteria were used as an initial screening approach, followed by phenotypic and molecular characterization of the recovered isolate^28^.

### Sample collection, preparation and fermentation

Twelve samples of naturally fermented cucumber were collected from local markets in Sohag Governorate, Egypt, as potential sources of EPS-producing microorganisms. Samples were aseptically transferred into sterile containers and transported under refrigerated conditions (4 °C) to the Bacteriology Laboratory, Department of Botany and Microbiology, Faculty of Science, Sohag University, for immediate processing.

For microbial enrichment, 10 g of each sample were suspended in 100 mL of sterile distilled water and incubated at 25–30 °C for 72 h under spontaneous fermentation conditions. This enrichment step was intended to promote the growth of diverse fermentative microbial populations, including potential EPS producers, prior to isolation and screening.

### Screening of EPS-producing isolates

Ten grams of each enriched cucumber sample were aseptically transferred into sterile 250-mL flasks containing 40 mL of MRS broth and incubated at 37 °C with shaking (150 rpm) for 24 h to facilitate microbial growth. The initial pH of the MRS medium was adjusted to 6.5 prior to sterilization. Following incubation, 0.25 mL aliquots were spread onto MRS agar plates and incubated at 37 °C for 24 h to obtain discrete colonies. MRS medium was employed as a broadly supportive medium for fermentative bacteria potentially associated with EPS production^29^.

Multiple bacterial isolates were recovered. Catalase testing was performed as a preliminary differentiation step to distinguish catalase-negative LAB-like isolates from catalase-positive bacteria. Among the recovered colonies, one catalase-positive isolate exhibiting a distinctly mucoid and reproducible colony morphology was selected for further characterization based on its pronounced EPS-associated phenotype.

Well-isolated colonies were subcultured to ensure purity and maintained on MRS agar at 4 °C for short-term storage and in MRS broth supplemented with 20% (v/v) glycerol at − 20 °C for long-term preservation. To enhance EPS synthesis, purified isolates were cultured on MRS agar supplemented with 10% (w/v) sucrose and incubated at 37 °C for 24 h.

Colonies displaying a mucoid, shiny, or sticky appearance were considered potential EPS producers. Preliminary EPS production was qualitatively assessed using the thread-formation test, in which a sterile inoculating loop was gently lifted from the colony surface; formation of a continuous viscous thread was interpreted as indicative of extracellular polysaccharide secretion^30^. This agar-based screening method was used solely as an initial qualitative indicator prior to subsequent physicochemical and spectroscopic characterization.

### Identification of the selected EPS-producing bacterial isolate

#### Morphological and biochemical characterization

Morphological and preliminary biochemical characterization of the selected isolate was performed to support its taxonomic placement. Gram staining was carried out according to standard microbiological procedures to determine cell morphology and Gram reaction^31^. Catalase activity was evaluated by adding 3% (v/v) hydrogen peroxide to freshly grown colonies and observing immediate bubble formation. Oxidase activity was determined using 1% (w/v) tetramethyl-p-phenylenediamine dihydrochloride reagent following established biochemical testing protocols^32^. A coagulation test using sterile plasma was performed as part of routine biochemical profiling. All assays were conducted in triplicate to ensure reproducibility.

#### Molecular identification by 16 S rRNA gene sequencing

Genomic DNA was extracted from the purified isolate using a standard bacterial DNA extraction protocol. The nearly full-length 16 S rRNA gene was amplified by PCR using the universal bacterial primers 27 F (5′-AGAGTTTGATCMTGGCTCAG-3′) and 1492R (5′-TACGGYTACCTTGTTACGACTT-3′), as originally described for bacterial systematics studies^31^. The PCR product (~ 1.4 kb) was verified by agarose gel electrophoresis and purified prior to sequencing. Sequencing was performed by SolGent Co. Ltd. (Daejeon, South Korea). The resulting 16 S rRNA gene sequence (1418 bp) was compared with reference sequences in the NCBI GenBank database using the BLASTn algorithm^33^.

Sequence similarity analysis revealed that strain HH shared ≥ 99.8% sequence identity with *Bacillus tequilensis*, clustering within members of the *Bacillus subtilis* species complex. It is well recognized that 16 S rRNA gene sequencing may not provide sufficient resolution to discriminate closely related species within the *Bacillus subtilis* species complex, and therefore species-level assignment based solely on this marker should be interpreted cautiously^34^. Based on the highest sequence similarity and phylogenetic clustering, the isolate was designated as *Bacillus tequilensis* strain HH, a member of the *Bacillus subtilis* species complex. The 16 S rRNA gene sequence was deposited in the GenBank database under accession number PX517619.

#### Phylogenetic analysis

The obtained 16 S rRNA gene sequence of strain HH was aligned with closely related reference sequences retrieved from the NCBI GenBank database using the Clustal W algorithm implemented in MegAlign software version 5.05 (DNASTAR Inc., Madison, WI, USA)^35^. Phylogenetic reconstruction was performed using the Neighbor-Joining method based on the aligned sequences. The reliability of the inferred tree topology was evaluated by bootstrap analysis with 1,000 replicates. *Escherichia coli* (NR_024570) was included as an outgroup to root the tree.

### Determination of Sucrose-hydrolyzing activity

Sucrose-hydrolyzing activity in culture supernatants was evaluated as an indicator of sucrose utilization under different environmental and nutritional conditions. *Bacillus tequilensis* strain HH, a member of the *Bacillus subtilis* species complex, was cultivated in MRS broth supplemented with varying sucrose concentrations (8–16%, w/v) and incubated at different pH values (5.0–8.0) and temperatures (25–45 °C) under aerobic and anaerobic conditions. Cultures were incubated for 24 h, after which samples were collected for analysis of extracellular sucrose-hydrolyzing activity.

Following incubation, cultures were centrifuged at 6,000 rpm for 10 min at 4 °C, and the resulting cell-free supernatant was used as a crude enzyme preparation. Sucrose-hydrolyzing activity was determined using sucrose as the substrate by quantifying the release of reducing sugars according to the dinitrosalicylic acid (DNS) method described by Miller^36^. The DNS assay has been widely employed for estimating glucansucrase and related sucrose-hydrolyzing enzyme activities based on reducing sugar release^37,38^. Absorbance was measured at 540 nm, and glucose was used as the standard to construct the calibration curve.

One unit (U) of sucrose-hydrolyzing activity was defined as the amount of enzyme required to release 1 µmol of reducing sugar (expressed as glucose equivalents) per minute under the assay conditions^36^. All experiments were performed in triplicate, and results were expressed as mean ± standard deviation (SD). Statistical analysis was carried out using one-way analysis of variance (ANOVA) followed by Tukey’s post hoc test, with statistical significance considered at *p* < 0.05. This assay provides an indirect measure of reducing sugar release and does not specifically confirm the activity of glucansucrase enzymes.

### Production and partial purification of exopolysaccharides (EPS)

*Bacillus tequilensis* strain HH, a member of the *Bacillus subtilis* species complex, was initially cultured in 10 mL of MRS broth at 37 °C for 24 h under static conditions to obtain active biomass. The pre-culture was subsequently transferred into 100 mL of fresh MRS broth and incubated for an additional 24 h.

For preparative EPS production, the culture was inoculated into 1 L of MRS broth supplemented with 10% (w/v) sucrose as the primary carbon source. Fermentation was conducted at 37 °C for 20 h under mild agitation (100 rpm). The initial pH of the medium was adjusted within the range of 5.0–6.5 to support bacterial growth under the selected production conditions^39^.

Following fermentation, cultures were centrifuged at 6,000 rpm for 15 min at 4 °C to remove bacterial cells. The resulting cell-free supernatant was subjected to ethanol precipitation as an initial recovery step. Briefly, three volumes of ice-cold 98% (v/v) ethanol were added to the supernatant, and the mixture was incubated at 4 °C to allow polysaccharide precipitation. The precipitate was collected by centrifugation, re-dissolved in distilled water, and re-precipitated with ethanol to reduce low-molecular-weight soluble components. No chromatographic purification, dialysis, or dedicated protein- or nucleic acid-removal procedures were performed at this stage. Accordingly, the recovered material was regarded as a partially purified EPS fraction and was used for subsequent physicochemical and spectroscopic characterization^40^. Ethanol precipitation is widely employed as an initial recovery approach for extracellular polysaccharides prior to advanced purification procedures^41^.

The precipitated EPS was freeze-dried (lyophilized) to constant weight. The crude yield (prior to re-precipitation) was determined gravimetrically. Recovery efficiency was calculated as (W₂/W₁) × 100, where W₁ represents the crude EPS yield and W₂ corresponds to the partially purified EPS yield. All determinations were performed in triplicate, and results were expressed as mean ± standard deviation (SD).

### Structural characterization of EPS (FTIR, ¹H NMR)

Fourier Transform Infrared (FTIR) spectroscopy was performed to identify the major functional groups present in the partially purified exopolysaccharide (EPS) fraction. Spectra were recorded using an Attenuated Total Reflectance (ATR) accessory on a Thermo Scientific Nicolet™ iS10 spectrometer over the range of 4000–500 cm⁻¹. The analysis was conducted following standard procedures commonly applied for structural assessment of microbial polysaccharides^42^.

Proton Nuclear Magnetic Resonance (¹H NMR) spectroscopy was carried out to obtain preliminary structural information regarding the recovered EPS fraction. Approximately 30 mg of the partially purified EPS was dissolved in deuterated dimethyl sulfoxide (DMSO-d₆). The ¹H NMR spectrum was recorded on a Bruker 400 MHz spectrometer at ambient temperature. Chemical shifts were expressed in parts per million (ppm) relative to the residual solvent signal. The use of ¹H NMR spectroscopy for structural analysis of carbohydrate polymers has been widely reported in the literature^43^.

It should be emphasized that the present NMR analysis was limited to one-dimensional ¹H NMR; therefore, definitive assignment of glycosidic linkages or branching patterns was not attempted in this study.

### Physicochemical and rheological characterization of EPS

Viscosity measurements of EPS solutions were performed using an Ubbelohde capillary viscometer at 25 ± 1 °C. Flow times were recorded for distilled water and for EPS solutions prepared at concentrations of 0.5, 1, 1.5, and 2% (w/v) in 0.9% (w/v) NaCl solution. All measurements were conducted in triplicate.

Relative viscosity (ηr) and specific viscosity (ηsp) were calculated according to standard capillary viscometry equations^44^:

$$\eta r{\text{ }} = {\text{ }}t{\text{ }}/{\text{ }}t_{0}$$

$$\eta sp = \eta r - 1$$

where t is the flow time of the EPS solution (s) and t₀ is the flow time of the solvent (s).

Reduced viscosity (ηred) was calculated as:

$$\eta red = \eta sp/C$$

where C is the polymer concentration expressed in g dL⁻¹.

Intrinsic viscosity [η] was estimated by extrapolating reduced viscosity values to zero concentration. The viscosity-average molecular weight (M_v_) was estimated using the Mark–Houwink–Sakurada equation:

$$\left[ \eta \right]{\text{ }} = {\text{ }}K{\text{ }} \times {\text{ }}M_{v}^{a}$$

Literature-reported Mark–Houwink constants for water-soluble glucan-type polysaccharides in aqueous solution at 25 °C were applied^45,46^(K = 1.0–1.6 × 10⁻⁴ dL g⁻¹; a = 0.70–0.85). The calculated molecular weight therefore represents an approximate viscometric estimation derived from intrinsic viscosity measurements^44^. This estimation assumes polymer behavior comparable to literature-reported glucan standards and does not constitute a direct molecular weight determination. The calculated M_v_ values do not correspond to absolute molecular weight measurements such as those obtained by size-exclusion chromatography (SEC-GPC).

To assess solution stability, the viscosity of EPS solutions was monitored over a 24 h period under identical experimental conditions.

All experiments were performed in triplicate, and results were expressed as mean ± standard deviation (SD).

###  Cytotoxicity and biocompatibility evaluation

#### Cell Lines and culture conditions

The in vitro cytocompatibility of the partially purified glucan-type exopolysaccharide (EPS) produced by *Bacillus tequilensis* strain HH was evaluated using two mammalian cell lines: WI-38 (human lung fibroblast) and Vero (African green monkey kidney epithelial) cells. Cells were cultured in RPMI-1640 medium supplemented with 10% fetal bovine serum (FBS), 1% penicillin streptomycin, and 1% L-glutamine, and maintained at 37 °C in a humidified atmosphere containing 5% CO₂ until reaching appropriate confluence.

Both WI-38 and Vero cell lines were obtained from the Cell Culture Collection Unit, Holding Company for Biological Products and Vaccines (VACSERA), Giza, Egypt. All experiments were conducted at the Genomics and Immunology Center (GIC), Faculty of Medicine, Cairo University, Egypt.

#### Cell viability assay

Cell viability was evaluated using the MTT colorimetric assay, which quantifies mitochondrial metabolic activity as an indirect indicator of viable cells^47^. WI-38 and Vero cells were seeded in 96-well plates at a density of 1 × 10⁵ cells/mL (100 µL per well) and allowed to adhere for 24 h under standard culture conditions. Cells were subsequently exposed to serial concentrations (31.25–1000 µg/mL) of the partially purified EPS.

Following 24 h of treatment at 37 °C in a humidified atmosphere containing 5% CO₂, 20 µL of MTT solution (5 mg/mL) was added to each well, and the plates were incubated for an additional 4 h to allow for formazan crystal formation. The resulting crystals were dissolved in 200 µL of dimethyl sulfoxide (DMSO), and absorbance was measured at 560 nm with a reference wavelength of 620 nm using a BioTek microplate reader.

Cell viability (%) was calculated relative to untreated control cells^48,49^. Dose–response curves were constructed, and IC₅₀ values were determined from nonlinear regression analysis of the concentration–response data. All experiments were performed in triplicate, and results were expressed as mean ± standard deviation.

#### Morphological observation

Following EPS treatment, cellular morphology was examined using an inverted phase-contrast microscope to monitor general morphological changes compared with untreated control cells. Representative fields were observed and documented under identical imaging conditions.

## Results

### Screening of EPS-producing isolates

Following sample enrichment under spontaneous fermentation conditions, twelve bacterial isolates were recovered from fermented cucumber samples. Preliminary phenotypic screening showed that eleven isolates were catalase-negative, whereas one isolate exhibited a catalase-positive reaction.

When cultured on MRS agar supplemented with sucrose, the catalase-positive isolate developed visibly mucoid colony morphology. During qualitative screening using the thread-formation test, the isolate produced a distinct viscous filament upon gentle lifting with an inoculating loop, indicating extracellular polymer production.

Based on the reproducibility of its mucoid phenotype and positive thread-formation response, this isolate was selected for subsequent phenotypic, molecular, and physicochemical characterization.

### Identification of the isolate

#### Morphological and biochemical characteristics

Microscopic examination revealed Gram-positive, rod-shaped bacterial cells occurring singly or in short chains. When cultivated on MRS agar supplemented with sucrose, the isolate formed opaque, mucoid colonies with a viscous surface appearance.

Biochemical characterization showed that the isolate was catalase-positive and oxidase-negative, with no coagulation observed in the plasma test. These phenotypic features are consistent with members of the genus *Bacillus* and distinguish the isolate from typical lactic acid bacteria, which are generally catalase-negative.

#### Molecular identification and phylogenetic analysis

Analysis of the nearly full-length 16 S rRNA gene sequence (1418 bp) showed that strain HH shared high sequence similarity (99.86–99.93%) and complete query coverage with members of the *Bacillus subtilis* species complex in the NCBI GenBank database, including *Bacillus tequilensis*, *Bacillus subtilis*, and closely related taxa.

To further support taxonomic assignment, the sequence was analyzed using the EzBioCloud database, which compares query sequences against curated type strain references. EzBioCloud analysis indicated that type strains of *Bacillus tequilensis* exhibited the highest sequence similarity to strain HH.

**Fig. 1:**
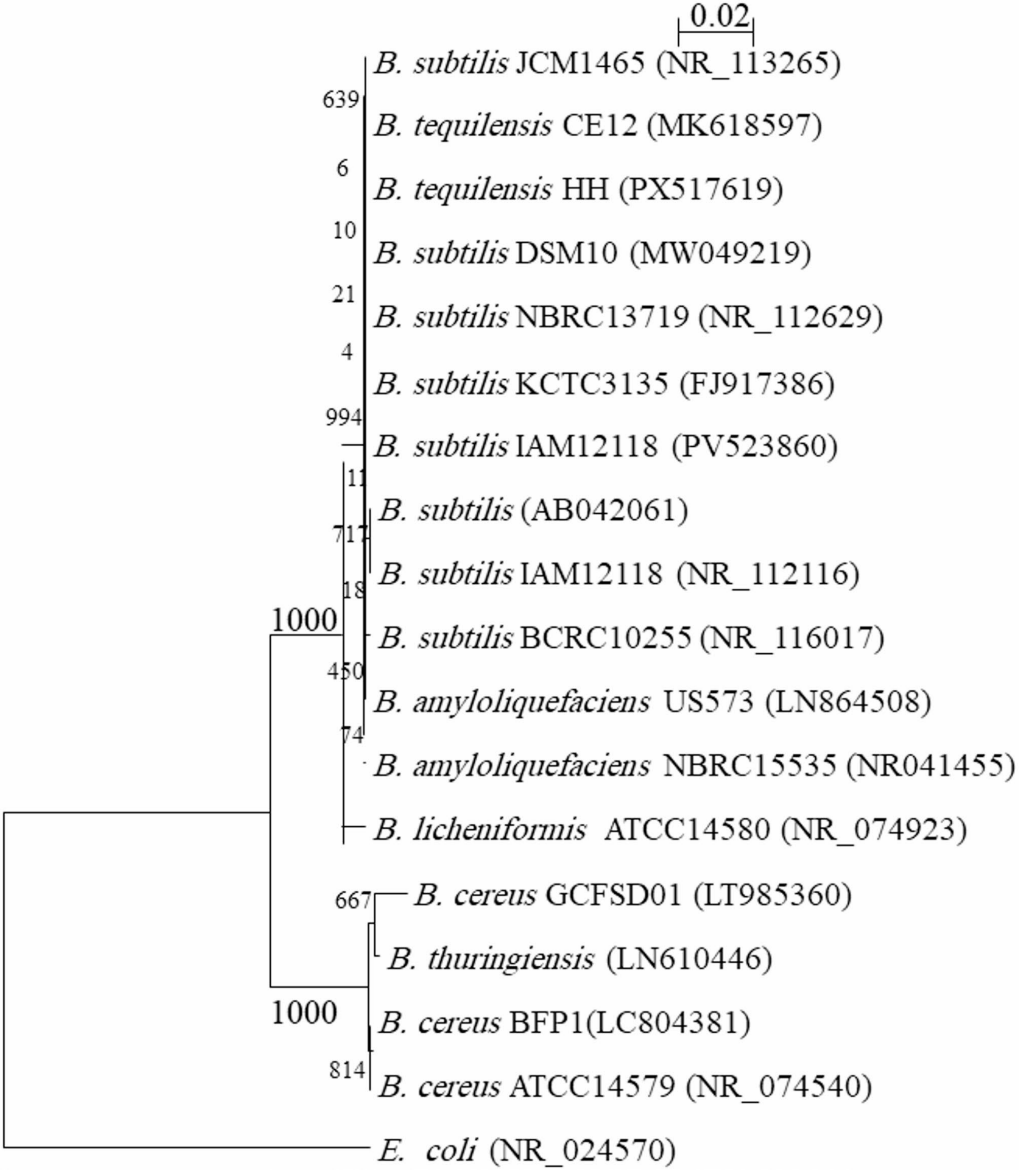
Phylogenetic tree based on 16 S rRNA gene sequences showing the position of *Bacillus tequilensis* strain HH (PX517619) among closely related members of the *Bacillus subtilis* species complex. The tree was constructed using the Neighbor-Joining method following Clustal W alignment. Bootstrap values (1,000 replicates) are shown at branch nodes. *Escherichia coli* (NR_024570) was used as an outgroup. Scale bar indicates 0.02 nucleotide substitutions per site.

Phylogenetic reconstruction based on 16 S rRNA gene sequences (Neighbor-Joining method with 1,000 bootstrap replicates) showed that strain HH clustered with reference strains of *Bacillus tequilensis* within the *Bacillus subtilis* species complex **(**Fig. 1**)**. However, due to the high sequence conservation among closely related taxa within this complex, species-level resolution based solely on 16 S rRNA gene analysis remains limited.

Accordingly, the isolate was designated as *Bacillus tequilensis* strain HH, a member of the *Bacillus subtilis* species complex, based on highest sequence similarity and phylogenetic clustering, while acknowledging the inherent limitations of 16 S rRNA gene-based discrimination within this group. The 16 S rRNA gene sequence was deposited in GenBank under accession number PX517619.

### Sucrose-hydrolyzing activity

Sucrose-hydrolyzing activity of *Bacillus tequilensis* strain HH was evaluated under varying physicochemical and nutritional parameters, including pH, sucrose concentration, temperature, aeration, and incubation time (Table 1). Enzymatic activity was significantly influenced by all tested factors (*p* < 0.05).

**Table 1:** Effect of growth parameters on sucrose-hydrolyzing activity of *Bacillus tequilensis* strain HH.

Parameter	Range Tested	Optimal Value	Activity (U/mL)
pH	3–11	6.0	0.0240 ± 0.0002
Sucrose concentration (%)	2–14	14	0.069 ± 0.0006
Temperature (°C)	25–45	37	0.048 ± 0.002
Aeration	Static vs. shaking (50 rpm)	Aerobic shaking (50 rpm)	2.018 ± 0.100
Incubation time (h)	6–72	24	1.783 ± 0.015

Values represent mean ± standard deviation of three independent experiments. Statistical significance was determined by one-way ANOVA followed by Tukey’s post hoc test (*p* < 0.05).

Among the tested pH values (3–11), maximum activity was observed at pH 6.0 (0.0240 ± 0.0002 U/mL). Increasing sucrose concentration (2–14%) resulted in a progressive increase in activity, reaching a maximum at 14% sucrose (0.069 ± 0.0006 U/mL). Temperature markedly affected enzyme activity, with the highest value recorded at 37 °C (0.048 ± 0.002 U/mL), followed by a decline at elevated temperatures.

With respect to aeration, activity under aerobic shaking at 50 rpm (2.018 ± 0.100 U/mL) was significantly higher than under static cultivation (1.682 ± 0.084 U/mL), as confirmed by one-way ANOVA (F = 19.234, *p* = 0.001) followed by Tukey’s post hoc test (*p* < 0.001).

Incubation time also significantly influenced enzyme production, with peak activity observed after 24 h (1.783 ± 0.015 U/mL), followed by a gradual reduction upon prolonged incubation.

These findings demonstrate that sucrose-hydrolyzing activity is modulated by environmental and nutritional conditions without implying a specific enzymatic mechanism. Absolute activity values are not directly comparable across parameters, as each factor was evaluated independently under its respective experimental framework.

### Production and recovery of EPS

Following cultivation of *Bacillus tequilensis* strain HH under the production conditions described in Sect.  2.5, extracellular polymeric material was detected in the culture supernatant. After removal of bacterial cells by centrifugation, ethanol precipitation yielded a whitish fibrous precipitate consistent with polymeric material.

The precipitated fraction was collected and subjected to re-dissolution in distilled water followed by re-precipitation with ethanol to reduce low-molecular-weight soluble components. The crude EPS yield (prior to re-precipitation) was 0.30 ± 0.01 g/100 mL of culture broth. After the re-precipitation step, the partially purified fraction yielded 0.22 ± 0.02 g/100 mL, corresponding to a recovery efficiency of 73.3%. The reported EPS yield represents crude recovery after partial purification and was not normalized based on total carbohydrate content; therefore, it should be interpreted as an approximate recovery value rather than an absolute production yield.

The recovered EPS was freeze-dried to obtain a stable powdered form and reconstituted in distilled water, yielding a viscous and water-soluble fraction. This behavior is consistent with the typical properties of microbial polysaccharides and supports its suitability for subsequent physicochemical and biological analyses.

### Structural characterization

#### FTIR spectroscopy

**Fig. 2:**
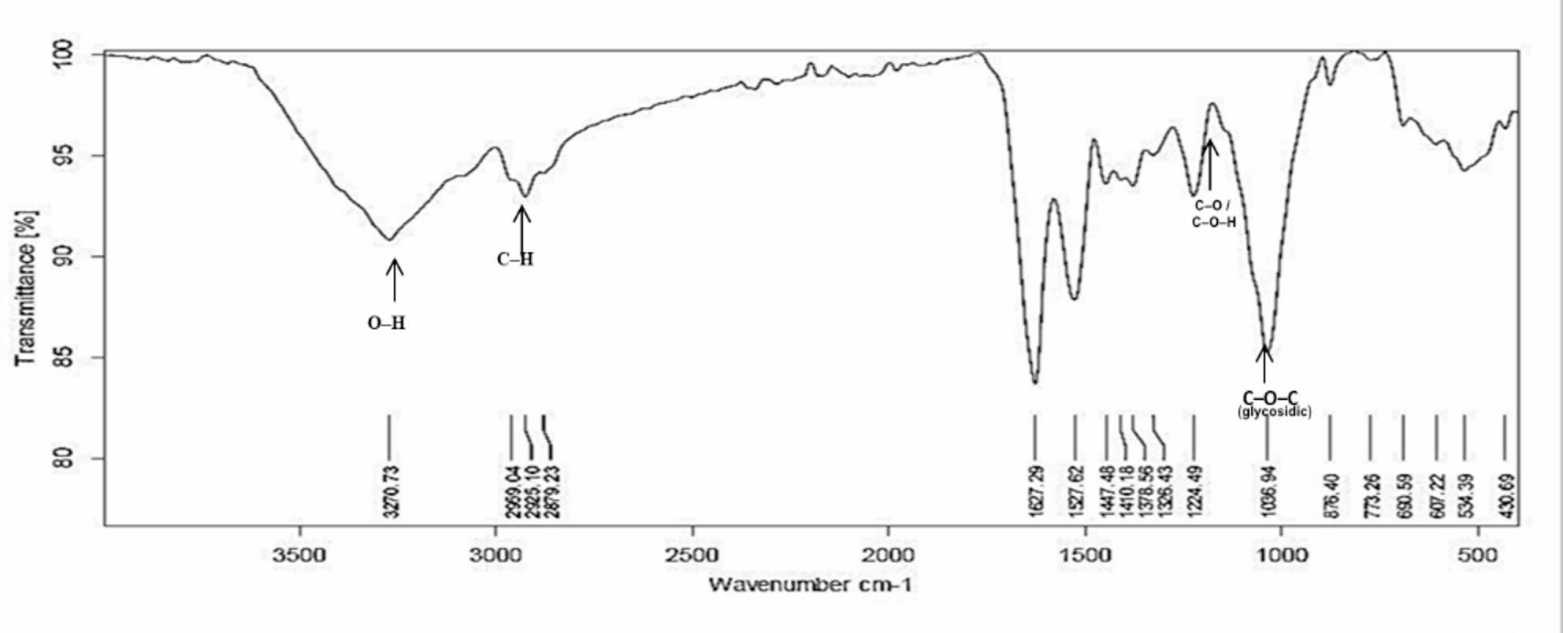
FTIR spectrum of the partially purified exopolysaccharide (EPS) produced by *Bacillus tequilensis* strain HH, showing absorption bands consistent with polysaccharide structures.

The FTIR spectrum of the partially purified exopolysaccharide (EPS) exhibited characteristic absorption bands typical of carbohydrate polymers (Fig. 2; Table 2). A broad absorption band observed around 3270 cm⁻¹ corresponds to O–H stretching vibrations, indicating the presence of hydroxyl groups involved in extensive hydrogen bonding, a common feature of polysaccharides.

**Table 2:** FTIR spectral analysis of the partially purified exopolysaccharide produced by *Bacillus tequilensis* strain HH.

Wavenumber (cm⁻¹)	Functional group	Vibration type	Assignment / interpretation	References
~ 3270	O–H	Stretching	Hydroxyl groups involved in hydrogen bonding, characteristic of polysaccharides	^42^
2929–2889	C–H	Stretching	Aliphatic C–H stretching vibrations of sugar residues	^50^
~ 1627	H–O–H	Bending	Bound water associated with polysaccharide structure	^43^
~ 1527	N–H / C–H	Bending	Possible contribution from residual protein traces or overlapping bending vibrations.	^50^
1440–1371	C–H	Bending	CH₂/CH₃ deformation vibrations	^51^
~ 1244	C–O	Stretching	C–O stretching associated with polysaccharide backbone	^43^
~ 1066	C–O–C / C–O–H	Stretching	C–O–C stretching of glycosidic bonds commonly associated with glycosidic linkages in glucan-type polysaccharides	^50^
876–560	C–H	Bending	Anomeric region and ring vibrations of sugar residues	^51^

Absorption bands in the region of 2929–2889 cm⁻¹ are attributed to C–H stretching vibrations of aliphatic groups present in sugar residues. The band detected near 1627 cm⁻¹ is associated with O–H bending vibrations, likely due to bound water molecules within the polymer matrix.

Signals observed in the range of 1527–1401 cm⁻¹ correspond to deformation vibrations of C–H and may also indicate minor contributions from carboxylate groups. The region between 1311 and 1242 cm⁻¹ is assigned to C–O stretching vibrations of the polysaccharide backbone.

A prominent peak at approximately 1066 cm⁻¹ is characteristic of C–O–C stretching vibrations of glycosidic linkages, representing a key structural feature of glucan-type polysaccharides. Additional bands in the region of 873–773 cm⁻¹ are attributed to ring vibrations and the anomeric region of sugar residues^50^.

#### ^1^H NMR spectroscopy

The ¹H NMR spectrum of the partially purified EPS revealed characteristic signals within the anomeric proton region (δ 5.1–5.4 ppm), which are typically associated with glucan-type polysaccharides. Multiple resonances observed in the range of δ 3.2–4.0 ppm correspond to ring protons of glucose residues, consistent with proton environments in carbohydrate backbones. A signal detected near δ 3.6 ppm is attributed to CH₂OH proton groups, which are commonly present in glucopyranosyl units.

These spectral features are consistent with previously reported glucan-type exopolysaccharides produced by *Bacillus* species^51^. Similar chemical shift distributions in the anomeric and ring proton regions have been reported in previous studies for microbial polysaccharides. Collectively, these signals support the presence of a glucan-type polysaccharide structure.

**Fig. 3:**
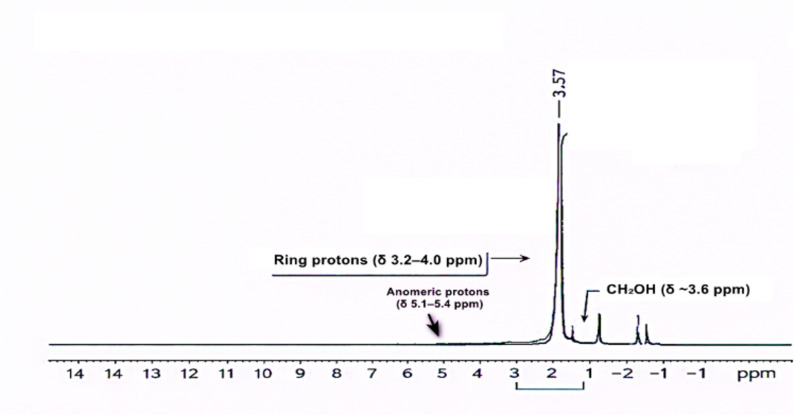
^1^H NMR spectrum of the partially purified exopolysaccharide (EPS) produced by *Bacillus tequilensis* strain HH.

However, due to the use of one-dimensional ¹H NMR analysis alone, definitive structural assignment of glycosidic linkage positions or branching patterns was not performed. Comprehensive structural elucidation would require advanced techniques such as 2D NMR spectroscopy and linkage analysis (Fig. 3).

### Physicochemical and rheological properties

Viscosity measurements of EPS solutions demonstrated a clear concentration-dependent increase in flow time across the tested range (0.5–2%, w/v). As presented in Table 3, both relative viscosity (ηr) and specific viscosity (ηsp) increased progressively with polymer concentration, indicating enhanced resistance to flow at higher polymer contents. This behavior is characteristic of dissolved macromolecular systems, where intermolecular interactions and chain entanglement govern hydrodynamic behavior.

**Table 3:** Flow time and viscosity parameters of EPS produced by *Bacillus tequilensis* strain HH at different concentrations.

Concentration (% w/v)	C (g dL⁻¹)		Flow time (s)		Relative viscosity (ηr)		Specific viscosity (ηsp)		Reduced viscosity (ηred, dL g⁻¹)	
Water (control)	—	29.0		—		—		—		
0.5	0.5	36		1.241		0.241		0.483		
1.0	1.0	42		1.448		0.448		0.448		
1.5	1.5	55		1.897		0.897		0.598		
2.0	2.0	67		2.310		1.310		0.655		

ηr = t/ t_0_ sp = ηr − 1; ηred = ηsp/C; C expressed in g dL⁻¹. All measurements were performed at 25 ± 1 °C.

Reduced viscosity (ηred) values were calculated for each concentration. Linear extrapolation of reduced viscosity at low polymer concentrations (0.5–1.0 g dL⁻¹) yielded an intrinsic viscosity [η] of approximately 0.52 dL g⁻¹. Application of the Mark–Houwink–Sakurada relationship, using literature-reported constants for water-soluble glucan-type polysaccharides, resulted in a viscosity-average molecular weight in the range of approximately 1.4 × 10⁴ to 1.1 × 10⁵ g mol⁻¹.

This molecular weight interval places the recovered EPS within a low-to-moderate molecular weight category relative to certain high-molecular-weight microbial polysaccharides described in previous studies. The observed rheological behavior is therefore consistent with soluble polymer chains exhibiting moderate hydrodynamic dimensions in aqueous solution.

It should be emphasized that these molecular weight values represent approximate viscometric estimations derived from intrinsic viscosity measurements and depend on the selected Mark–Houwink parameters. They do not constitute absolute molecular weight determinations obtainable through chromatographic methods such as size-exclusion chromatography (SEC-GPC).

**Fig. 4:**
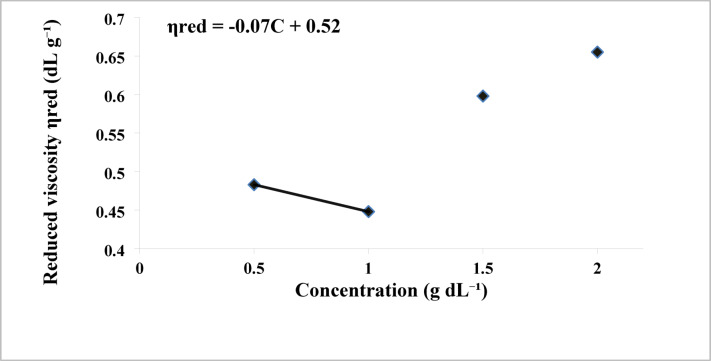
Reduced viscosity (ηred) as a function of EPS concentration (0.5–2.0%, w/v) in 0.9% NaCl at 25 ± 1 °C. The linear regression shown was derived from low-concentration data (0.5–1.0 g dL^-1^) for intrinsic viscosity estimation.

The relationship between EPS concentration and relative viscosity is illustrated in Fig. 4, demonstrating progressive thickening behavior with increasing polymer concentration.

### Cytotoxicity and cell viability

**Fig. 5:**
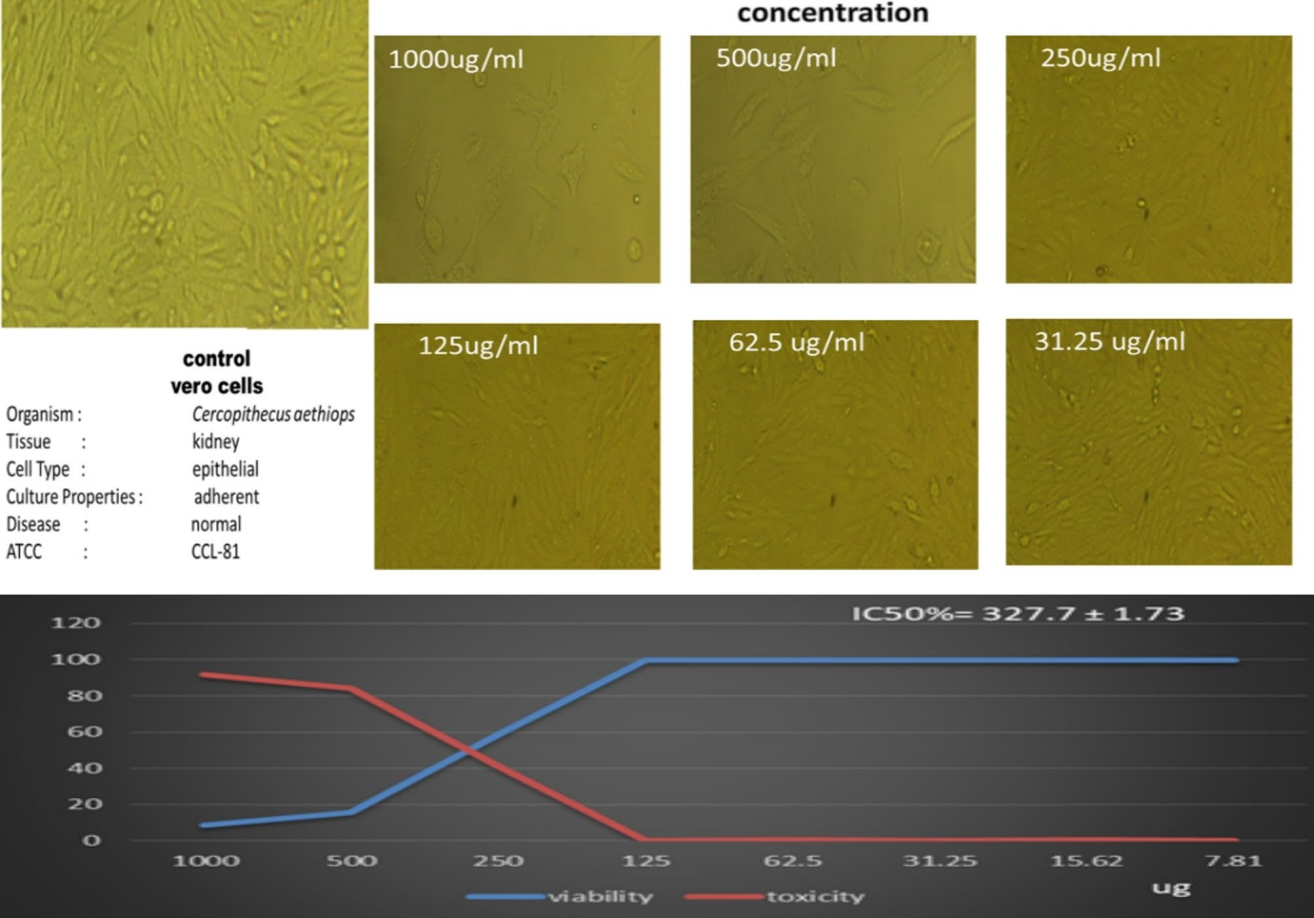
Dose–response curve of the exopolysaccharide (EPS) produced by *Bacillus tequilensis* strain HH in Vero cells. Cells were exposed to increasing concentrations of EPS (31.25–1000 µg/mL) for 24 h, and cell viability (%) was determined using the MTT assay. The IC_50_ value, calculated by nonlinear regression analysis, was 327.7 ± 1.73 µg/mL.

**Fig. 6:**
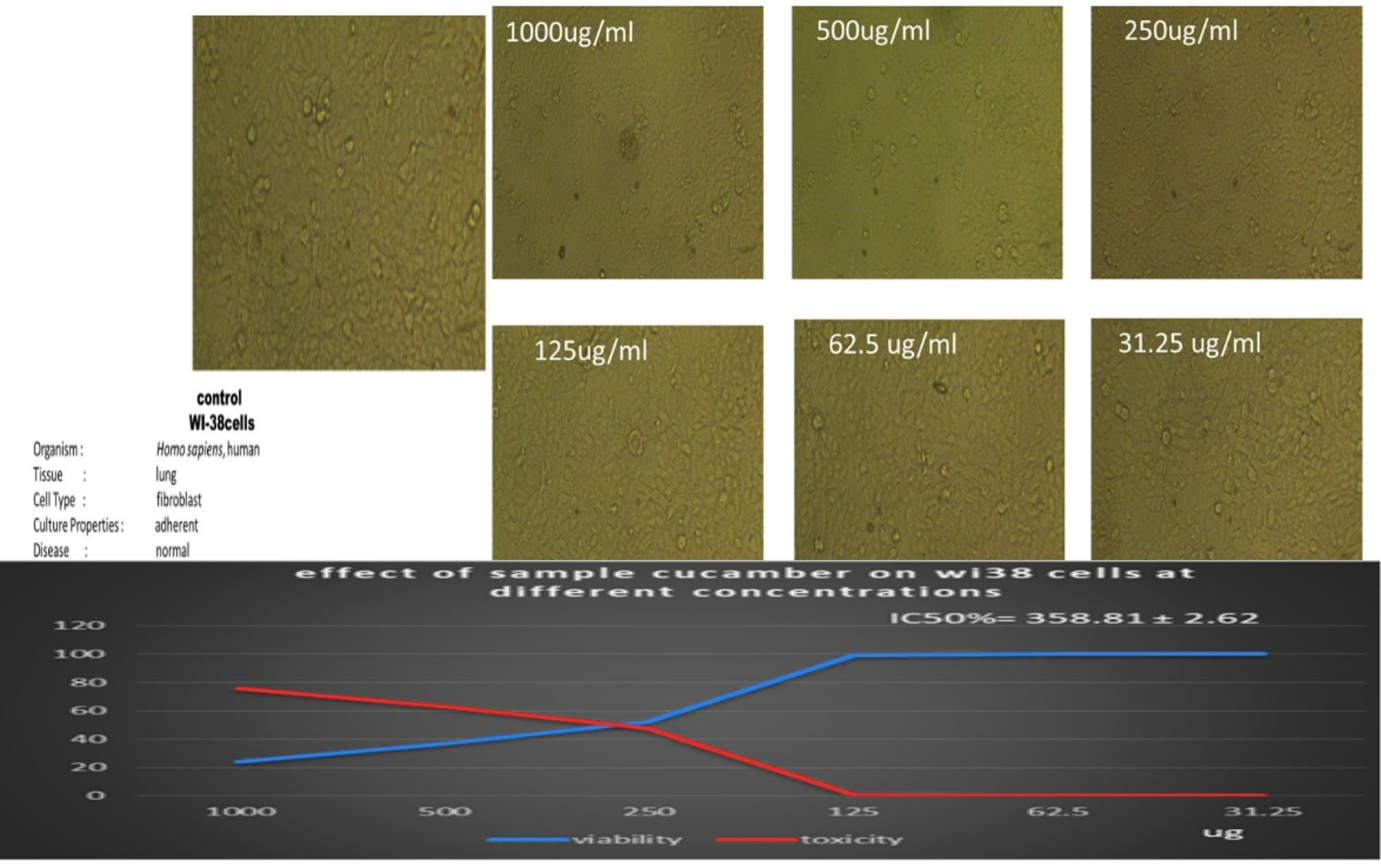
Dose–response curve of the exopolysaccharide (EPS) produced by *Bacillus tequilensis* strain HH in WI-38 cells. Cells were exposed to increasing concentrations of EPS (31.25–1000 µg/mL) for 24 h, and cell viability (%) was determined using the MTT assay. The IC_50_ value, calculated by nonlinear regression analysis, was 358.8 ± 2.62 µg/mL.

Cell viability following exposure to the exopolysaccharide (EPS) produced by *Bacillus tequilensis* strain HH was evaluated in Vero and WI-38 cell lines using the MTT assay. After 24 h of treatment, cell viability remained above 95% at low EPS concentrations (31.25–125 µg/mL), with no statistically significant difference compared to untreated control cells (*p* > 0.05) (Table 4). The corresponding dose–response curves are presented in Figs. 5 and 6.

**Table 4:** In vitro viability of Vero and WI-38 cells following 24 h exposure to the partially purified EPS produced by *Bacillus tequilensis* strain HH, as determined by the MTT assay.

Cell line	Concentration (µg/mL)	Mean O.D ± SD	Viability (%)
Vero	Control	0.716 ± 0.001	100.0
	31.25	0.716 ± 0.001	99.95
	62.5	0.713 ± 0.001	99.53
	125	0.715 ± 0.001	99.91
	250	0.421 ± 0.003	58.80
	500	0.111 ± 0.004	15.55
	1000	0.060 ± 0.001	8.43
WI-38	Control	0.743 ± 0.002	100.0
	31.25	0.743 ± 0.002	100.0
	62.5	0.743 ± 0.002	100.0
	125	0.739 ± 0.002	99.42
	250	0.389 ± 0.002	52.40
	500	0.273 ± 0.007	36.79
	1000	0.180 ± 0.005	24.27

IC_50_ values (mean ± SD) were 327.7 ± 1.73 µg/mL for Vero cells and 358.8 ± 2.62 µg/mL for WI-38 cells. Values are expressed as mean ± SD of three independent experiments. Cell viability (%) was calculated relative to untreated control cells.

At concentrations ≥ 250 µg/mL, a concentration-dependent reduction in metabolic activity was observed in both cell lines. At 250 µg/mL, cell viability decreased to 58.8% in Vero cells and 52.4% in WI-38 cells. Further reductions were detected at higher concentrations, reaching 15.6% and 36.8% viability at 500 µg/mL, and 8.4% and 24.3% at 1000 µg/mL in Vero and WI-38 cells, respectively.

The calculated half-maximal inhibitory concentration (IC_50_) values, determined by nonlinear regression analysis, were 327.7 ± 1.73 µg/mL for Vero cells and 358.8 ± 2.62 µg/mL for WI-38 cells, indicating concentration-dependent effects on cellular metabolic activity at elevated EPS levels.

Microscopic examination revealed no evident morphological alterations in either cell line at EPS concentrations up to 125 µg/mL. At higher concentrations, reduced cell density and partial cell rounding were observed, consistent with decreased metabolic activity measured by the MTT assay.

## Discussion

This study provides preliminary insight into the isolation of an exopolysaccharide-producing bacterium, identified as *Bacillus tequilensis* strain HH, from naturally fermented cucumber, along with an initial physicochemical characterization of the produced extracellular polymer under sucrose-rich conditions. Although members of the *Bacillus subtilis* species complex are not traditionally considered predominant producers of glucan-type exopolysaccharides in fermented foods, emerging evidence indicates that certain environmental isolates within this group possess inducible polysaccharide biosynthetic capabilities under specific nutritional conditions^52,53^. These findings further support the growing recognition of *Bacillus* species as potential contributors to exopolysaccharide production in diverse ecological niches, including fermented food systems^21,27^.

In this context, taxonomic identification of strain HH was primarily based on 16 S rRNA gene sequence analysis, which revealed high sequence similarity (99.86–99.93%) to multiple members of the *Bacillus subtilis* species complex. However, it is well documented that interspecies sequence similarity within this complex frequently exceeds 99%, thereby limiting the discriminatory resolution of 16 S rRNA gene analysis at the species level. Consequently, genome-based approaches such as average nucleotide identity (ANI) analysis or whole-genome sequencing are generally recommended for definitive species delineation within this taxonomic group^54^. These considerations highlight that the current taxonomic assignment should be interpreted with caution and may benefit from further genome-level confirmation. Consistent with this, comparison with curated type strain databases (EzBioCloud) and phylogenetic clustering analyses supported the assignment of the isolate to *Bacillus tequilensis*. Nevertheless, the limitations of 16 S-based identification within the *Bacillus subtilis* species complex remain, and future genome-level analyses would provide improved taxonomic resolution and confirmation^52^.

The exopolysaccharide recovered by ethanol precipitation exhibited spectroscopic features consistent with a glucan-type polymer, as indicated by FTIR and ¹H NMR analyses. The FTIR profile showed characteristic carbohydrate-related absorption bands, while the ¹H NMR spectrum revealed signals within the anomeric and sugar ring proton regions, in agreement with previously reported glucan-type exopolysaccharides^42^. Although FTIR and one-dimensional ¹H NMR provide useful preliminary structural information supporting the polysaccharide nature of the recovered EPS, these techniques alone are insufficient for definitive determination of glycosidic linkage positions, branching patterns, or full conformational architecture^43,57^. Accordingly, the polymer is conservatively described as a glucan-type EPS. This classification is based on spectroscopic indications and should not be considered a definitive compositional or structural confirmation. Detailed structural elucidation would require additional approaches, including monosaccharide composition analysis, linkage analysis, ¹³C NMR, two-dimensional NMR spectroscopy, and chromatographic fractionation^43,57^.

The EPS yield reported in this study represents crude recovery following partial purification and was not normalized based on total carbohydrate content, which may influence direct quantitative comparison with previously reported studies. Accordingly, the reported values should be interpreted as indicative rather than directly comparable production yields. This methodological limitation should be considered when interpreting yield values, as variations in extraction, purification, and quantification approaches can significantly affect reported EPS production levels across studies.

In parallel, sucrose-hydrolyzing activity detected in the culture supernatant reflects active carbohydrate metabolism under the applied growth conditions and may be associated with polysaccharide biosynthesis. However, no direct enzymatic or genetic evidence linking sucrose hydrolysis to EPS biosynthesis was established in the present study. Such metabolic capabilities have been widely reported in members of the *Bacillus subtilis* species complex, which are known to possess diverse carbohydrate-active enzyme systems involved in polysaccharide production and modification^22,27,55^. However, the detection of reducing sugars alone does not provide direct evidence of specific enzyme activity, such as dextransucrase-mediated glucan synthesis. Therefore, in the absence of gene-level or enzyme-specific characterization, no definitive conclusions can be drawn regarding the underlying biosynthetic mechanism of the produced EPS.

The rheological behavior of the recovered EPS was characterized by concentration-dependent increases in viscosity in aqueous solution, reflecting typical macromolecular interactions and chain entanglement phenomena commonly observed in microbial polysaccharides. Such behavior is indicative of polymer chain overlap and intermolecular interactions, which contribute to the formation of structured viscous systems. The estimated molecular weight range (10^4^–10^5^ g mol⁻¹) is lower than that reported for high-molecular-weight dextrans produced by *Leuconostoc* species^37,56^, yet remains consistent with values reported for several bacterial exopolysaccharides exhibiting moderate hydrodynamic dimensions^45^.

These characteristics support the classification of the recovered polymer as a soluble microbial polysaccharide of low-to-moderate molecular weight, which may influence its functional and rheological performance in solution. Biological evaluation demonstrated high cell viability at lower EPS concentrations, with a concentration-dependent reduction in metabolic activity observed at higher levels. Similar dose-dependent responses have been reported for microbial exopolysaccharides, where increased concentrations may affect cellular metabolic activity without necessarily inducing specific cytotoxic mechanisms. Under the experimental conditions applied, these findings suggest a concentration-dependent cellular response rather than direct cytotoxicity^47^.

Overall, the findings of the present study are consistent with previous reports on glucan-type exopolysaccharides produced by *Bacillus* species, particularly with respect to their spectroscopic features and solution behavior^50,52^. However, the current work extends these observations by providing preliminary insight into EPS production by a member of the *Bacillus subtilis* species complex isolated from a fermented vegetable system, which remains relatively underexplored.

Despite these findings, the study is limited by the use of preliminary analytical approaches and the absence of detailed structural and genomic analyses. Future investigations should therefore focus on comprehensive structural elucidation, enzyme characterization, and genome-based analysis to enable a more precise understanding of the biosynthetic pathways and functional properties of the produced exopolysaccharide.

### Limitations

This study provides a preliminary characterization of a glucan-type exopolysaccharide produced by *Bacillus tequilensis* strain HH. Nevertheless, comprehensive structural elucidation of the polymer was beyond the scope of this study. Advanced analytical approaches, including ¹³C NMR spectroscopy, two-dimensional NMR analysis, monosaccharide composition profiling, and glycosidic linkage determination, are generally required for definitive structural resolution of polysaccharides, as described in detailed structural characterization studies^57^. Future studies should include accurate quantification of total carbohydrate content using standard methods such as the phenol–sulfuric acid assay to better evaluate EPS yield and purity.

The EPS preparation was obtained through ethanol precipitation without chromatographic fractionation or analytical verification of complete protein and nucleic acid removal; therefore, it should be considered partially purified. In addition, the sucrose-hydrolyzing activity detected using the DNS assay reflects the release of reducing sugars and does not constitute direct evidence of dextransucrase-mediated polymer synthesis.

Furthermore, the reported molecular weight represents a viscosity-average hydrodynamic estimation calculated using literature-derived Mark–Houwink parameters. As such, it should be interpreted as an approximate value rather than an absolute molecular weight determination obtainable through chromatographic techniques such as size-exclusion chromatography.

## Conclusion

This study reports the isolation of an exopolysaccharide-producing bacterium identified as *Bacillus tequilensis* strain HH from naturally fermented cucumber and presents a preliminary characterization of the produced polymer. The results indicate that non-lactic acid bacteria associated with fermented vegetable ecosystems may represent additional sources of extracellular polysaccharides.

Spectroscopic and rheological analyses suggest that the recovered polymer is consistent with a glucan-type exopolysaccharide based on preliminary spectroscopic evidence exhibiting concentration-dependent solution behavior typical of microbial polysaccharides. In vitro evaluation demonstrated high cellular metabolic activity at lower concentrations, with a dose-dependent reduction observed at higher levels.

Although comprehensive structural elucidation and genome-level analyses were beyond the scope of this study, the findings are consistent with previously reported glucan-type exopolysaccharides and provide a basis for future investigations focused on detailed structural characterization, genomic analysis, and functional evaluation of EPS produced by *Bacillus tequilensis* in fermented food systems.

## Supplementary Information

Below is the link to the electronic supplementary material.

Supplementary Material 1

Supplementary Material 2

## Data Availability

The 16 S rRNA gene sequence generated in this study has been deposited in the NCBI GenBank database under accession number PX517619 (Bacillus tequilensis strain HH). All other data supporting the findings of this study are available from the corresponding author upon reasonable request.
