# Supplementary figures and images for: Isolation and preliminary characterization of a glucan-type exopolysaccharide produced by Bacillus tequilensis strain HH from Egyptian fermented cucumber
Source: Sci Rep. 2026 Jul 30;16:23655. doi: 10.1038/s41598-026-62841-4 (PMC13424139; doi:10.1038/s41598-026-62841-4)

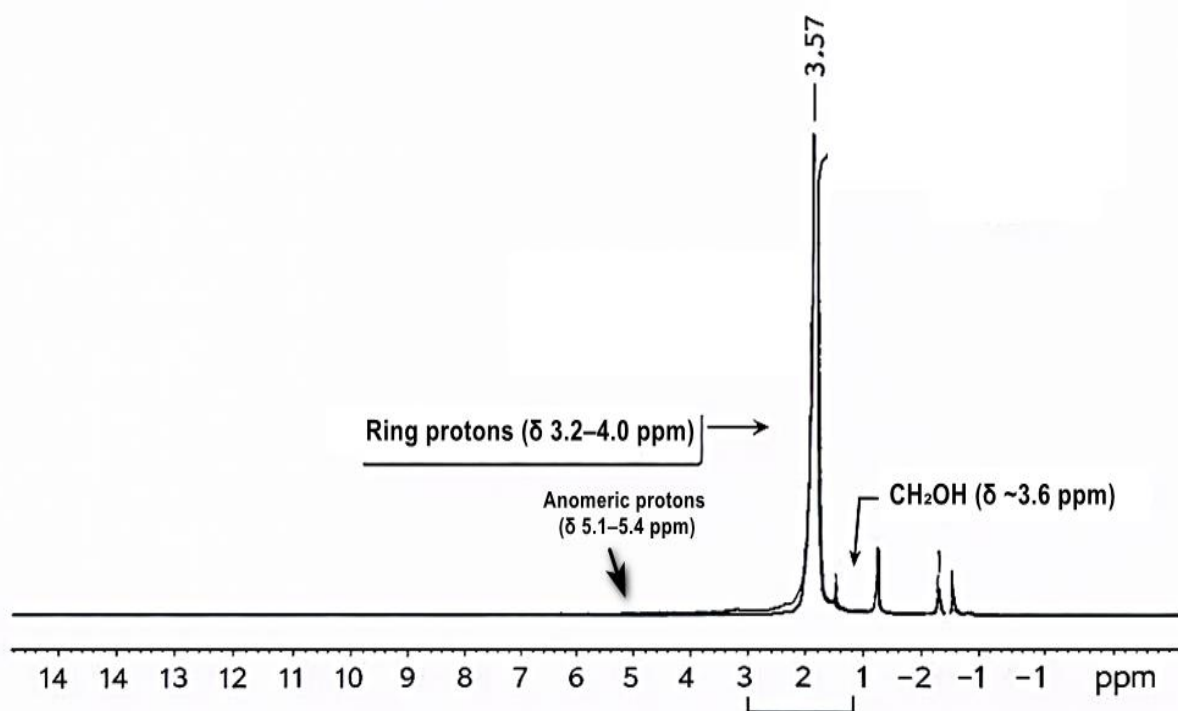

Supplement: Supplementary file 1 — Supplementary Material 1 [file 41598_2026_62841_MOESM1_ESM.pdf]

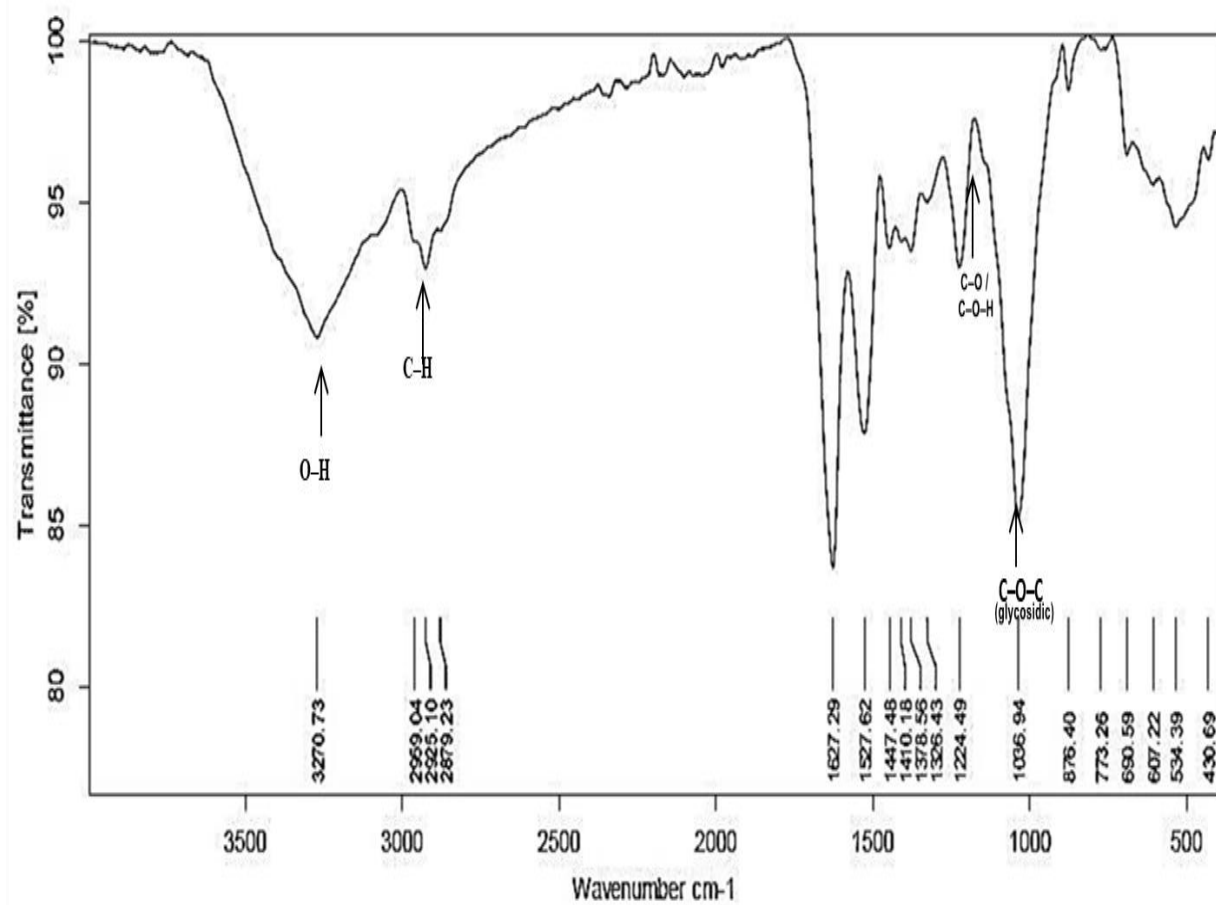

Supplement: Supplementary file 2 — Supplementary Material 2 [file 41598_2026_62841_MOESM2_ESM.pdf]
